# The effect of probiotic supplementation on performance, inflammatory markers and gastro‐intestinal symptoms in elite road cyclists

**DOI:** 10.1186/s12970-021-00432-6

**Published:** 2021-05-17

**Authors:** Chen Schreiber, Snait Tamir, Ron Golan, Ayelet Weinstein, Yitzhak Weinstein

**Affiliations:** 1 Department of Nutritional Sciences, Faculty of Science and Technology, Tel Hai Academic College , 1220800 Tel Hai, Israel; 2 Ziv Medical Center, Zefat, Israel

**Keywords:** Cyclists, Cycling performance, Gastrointestinal symptoms, Gastrointestinal integrity, Probiotics, Probiotic supplementation

## Abstract

**Background:**

Elite athletes may suffer from impaired immune function and gastro-intestinal (GI) symptoms, which may affect their health and may impede their performance. These symptoms may be reduced by multi-strain probiotic supplementation. Therefore, the aim of the current study is to examine the effects of probiotic supplementation on aerobic fitness characteristics, inflammatory markers and incidence and severity of GI symptoms in elite cyclists.

**Methods:**

Twenty-seven male cyclists, ranked elite or category 1 level competitions, were randomly assigned to a multi-strain probiotic-supplemented group (E, *n* = 11) or placebo group (C, *n* = 16). All participants visited the laboratory at the beginning of the study and following 90 d of supplementation/placebo. Prior to testing, all participants completed a GI symptoms questionnaire and underwent physical and medical examination, and anthropometric measurements. Venous blood was drawn for inflammatory markers analysis. The cyclists then underwent maximal oxygen consumption (VO_2_max) test and time-to-fatigue (TTF) test at 85 % of maximal power, 3 h following the VO_2_max test. All testing procedures were repeated after 90 d of probiotic / placebo treatment (double blind design).

**Results:**

Lower incidence of nausea, belching, and vomiting (*P* < 0.05) at rest, and decreased incidence of GI symptoms during training were found in E group vs. C Group, respectively (∆GI -0.27 ± 0.47 % vs. 0.08 ± 0.29 %, *P* = 0.03), no significant changes were observed in the incidence of total overall GI symptoms (∆GI -5.6 ± 14.7 % vs. 2.6 ± 11.6 %, *P* = 0.602) Mean rate of perceived exertion (RPE) values during the TTF were lower in E group (∆RPE: -0.3 ± 0.9 vs. 0.8 ± 1.5, *P* = 0.04). No significant changes were measured between and within groups in VO_2_max and TTF values, mean levels of C-reactive protein (CRP), IL-6-and tumor necrosis factor alpha (TNFα) values following treatment.

**Conclusions:**

Probiotics supplementation may have beneficial effects on GI symptoms in elite cyclists. Future studies, using higher doses and during different training seasons, might help understanding the effects of probiotic supplementation on elite athletes’ health and performance.

**Trial registration:**

NIH *clinicaltrial.gov* #NCT02756221 Registered 25 April 2016.

## Introduction

High physiological demands endured by elite cyclists who undergo intensive and prolonged training and competitions lead to numerous health-related side effects, including gastrointestinal (GI) discomfort and symptoms [1–3], which include upper GI (UGI) (nausea, belching, heartburn, chest pain and vomiting), and lower GI (LGI) symptoms (cramps, bloating, urge to defecate, defecation, diarrhea, flatulence and side ache). UGI and LGI symptoms have been reported by 46 and 54 % of elite cyclists during training, and even higher prevalence was reported during competition (53 and 60 %, respectively) [1, 4]. The etiology of these symptoms is commonly related to poor blood flow to splanchnic organs in response to increasing exercise intensity, which results in gut hypoperfusion and increased gut permeability [3, 5]. The damage to the gut integrity may lead to bacteria and bacterial toxin (i.e., lipopolysaccharide, LPS) translocation from the intestinal lumen into the blood circulation, which may induce a systemic cytokine response and inflammation and interfere with athletic stamina during sub-maximal efforts [3, 4, 6]. Strategies to minimize GI injury and inflammation during exercise may help reduce abdominal distress and impairments in the uptake of fluid, electrolytes, and nutrients thereby improving athletic health, performance and recovery [4, 7]. Some of these strategies include nutritional alterations such as consuming multi transportable carbohydrates during training, thus lowering dietary fibers content before and during exercise, and probiotic supplementation [4, 7, 8].

Increased attention has recently been given to probiotic supplementation using single or multi-strain products as a potential remedy for improving health and athletic performance in athletes undergoing high intensity training [7, 9, 10]. Probiotics consist of bacteria, especially lactic-acid producing bacteria, which are commercially available in capsules, as a powder or in selected dairy products such as fermented milk or yoghurt [8, 9]. They have potential health benefits, generally by improving or restoring the gut flora and demonstrating immune modulating capabilities [8]. Recent evidence suggests a relationship between the composition of intestinal microbiota and exercise, proposing that changes in the gut microbiota makeup may improve physical performance [6, 11]. Thus, probiotics supplementation used as means of improving gut microbiota function may have also added beneficial effects to athlete’s overall health [7, 11].

Various mechanisms have been proposed to explain these changes induced by probiotics, including the increase of mucus secretion and immunoglobulin A in the intestine, increase in tight junction stability between intestinal epithelial cells, and prevention of pathogenic bacteria excess growth by competing for binding sites on intestinal epithelial walls [7, 11, 12].

Interleukin 6 (IL-6) is a cytokine secreted from numerous tissues in response to probiotic supplementation and exercise and is commonly used as an inflammation marker [13–15]. While in some cases IL-6 increases CRP production in the liver, it may also inhibit the secretion of Tumor necrosis factor α (TNF-α) [16, 17]. Since TNF-α causes an increase in intestinal epithelial tight junction permeability [18], IL-6 modulation might influence gut health and athletic performance in fatigued athletes [19, 20].

Several studies have shown that probiotics supplementation could improve immune function in fatigued athletes [7, 21] and reduce upper respiratory tract illness (URTI) [22], GI symptoms [7, 11, 12] and gut permeability [15, 21]. However, only few studies have examined the effect of probiotics supplementation on athletic performance [9, 12]. In most studies multi-strain probiotics were more efficient in achieving these goals compared to single strain probiotics supplementation [7, 12], with 12 weeks being a common long term supplementation period. Supplementation of a single-strain probiotic (*Lactobacillus plantarum* at 2.05 × 10^8^ and 1.03 × 10^9^ CFU/kg/day) to mice lead to a significant decrease in body mass, increased muscle mass, increased forelimb grip strength and swimming endurance (time to exhaustion with added tail weight of 5 %), and decreased levels of serum lactate, ammonia, creatine kinase, and glucose following acute exercise (15 min swimming challenge) [23]. Time to fatigue (at 85 % of lactate threshold intensity) of young trained runners in hot conditions (35 °C, 40 % RH) was improved following 4 wk of multi-strain probiotics supplementation (45 billion CFU/day of *Lactobacillus*, *Bifidobacterium* and *Streptococcus* strains) compared with placebo [6]. Time to fatigue (at 85 % of VO_2_max) of young, healthy, non-athlete active participants was improved following 6 weeks of single strain (30 and 90 billion CFU/day of *Lactobacillus plantarum* TWK10) probiotics supplementation with dose dependent differences compared with placebo [24]. Supplementation with a single strain probiotic (12 billion CFU/day of *Lactobacillus fermentum*) for 4 wk lead to a non-significant rise in VO_2_max in trained runners compared to placebo [25]. Huang et al. [26] showed longer running durations during a VO_2_max ramp test in trained triathletes after *Lactobacillus plantarum* PS128 supplemetaion (30 billion CFU/day), but with no effect on VO_2_max values.

To the best of our knowledge there are no published studies that have directly investigated the effect of a long-term (e.g., > 60 d) multi-strain probiotic supplementation on elite cyclists’ health and performance. Hence, the aim of the current study was to identify potential health and physical performance benefits conferred by probiotic supplementation in elite cyclists by testing the effect of a multi-strain probiotic supplementation for 90 d on the cyclists’ GI symptoms, body composition, inflammatory markers and examine possible effects on maximal aerobic power (VO_2_max) and on time to fatigue.

## Methods

### Participants

Thirty male elite cyclists aged 19–40 y volunteered to participate in the study, which was approved by the Helsinki Ethics Committee of Ziv Medical Center, Zefat, Israel (# 0075**-**15**-**Ziv). Before the study began, its purpose and objectives were carefully explained to the participants, before they signed informed consents form. Three cyclists of the experimental group dropped out during the first month of the study (see below). Participants’ characteristics (*n* = 27) are shown in Table 1. Inclusion criteria necessitated that all cyclists competed at an elite or category 1 level competitions and continued with their normal training routine throughout the study duration. The cyclists were not limited in their training capacity due to illnesses or any other medical condition. The participants did not consume antibiotics or probiotic supplements, medications or ergogenic supplements in the 3 months preceding the study, and throughout the study duration. The study took place during the fall and early mild Mediterranean winter weather conditions.

**Table 1:** Anthropometric and fitness characteristics of the participants at the beginning of the study. Data are presented as mean±SD

	**All Participants (***N*=27**)**	**Experimental** **(***N*=11**)**	**Control** **(***N*=16**)**	***p*** value*
**Age, **y	28.3±5.6	25.9±4.6	29.5±6.2	0.12
**Weight, **kg	71.7±7.3	71.3±8.9	72.0±6.2)	0.82
**Height, **cm	176.4±5.2	178.1±5.5	175.1±4.8	0.14
**BMI, **kg/m^2^	23.2±2.2	22.6±2.7	23.5±1.9	0.33
**Body fat, **%	13.8±3.7	12.1±4.3	14.9±2.8*	0.05*
**Weekly training, **h	13.4±4.4	13.3±4.9	13.5±3.9	0.91

* *p*<0.05 Experimental vs. Control group (2-tailed independent t test)

### Study design and protocol

**Fig. 1:**
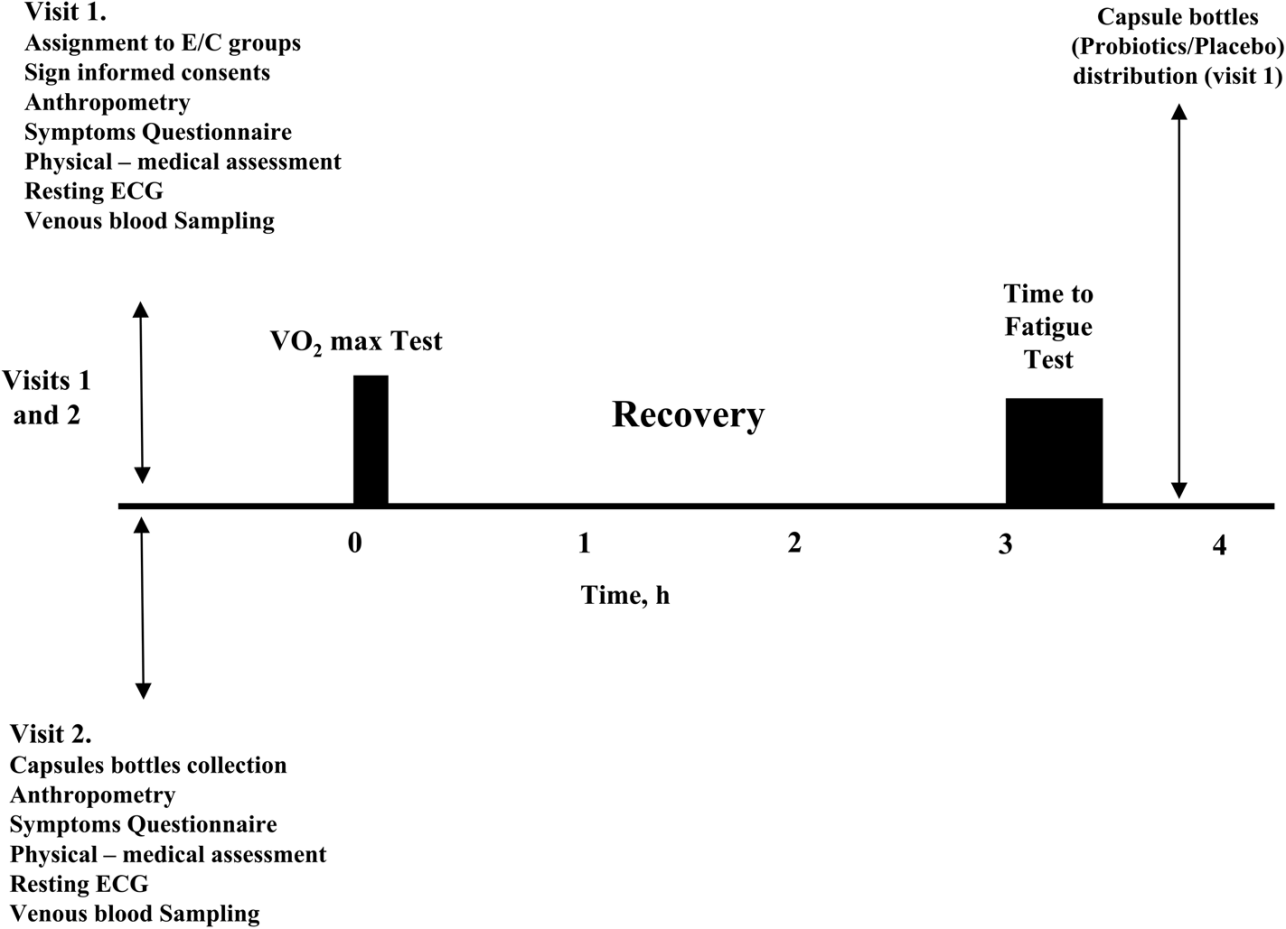
Test protocol for the experimental (E) and control (C) groups

The study followed a randomized, double-blind, two-arm, placebo-controlled trial design (see Fig. 1). Participants were randomized (as explained below), and underwent two session of laboratory tests, before and following 90 d of probiotic / placebo intervention. The cyclists were instructed to rest and refrain from strenuous activity for at least 24 h before the scheduled laboratory tests. The participants filled an online questionnaire to assess the frequency and severity of their GI symptoms prior to, during and after training and competitions.

During their first visit, the cyclists were randomly assigned into two groups: experimental (E) group (*n* = 11) and control (C) group (*n* = 16). E group participants received a 90 d supply of probiotic supplement capsules (see below), while C group received the same number of placebo capsules identical in shape and color to the probiotic capsules. All participants were instructed to begin consuming one capsule per day on the following day. During the first visit, baseline values were also recorded. Cyclists underwent anthropometric measurements and venous blood sampling for resting inflammatory markers analysis. The participants then underwent a series of tests which included a cardio-pulmonary exercise test (CPET) during which maximal oxygen consumption (VO_2_max), and the ventilatory threshold (VTh) were determined. Following 3 h rest, the participants performed a time-to-fatigue (TTF) test at 85 % of their maximal power (POmax) attained during the VO_2_max test. All tests were performed on a constant-power cycle ergometer. Following the 90 d supplementation/placebo period, the participants reported to the laboratory for a second series of tests identical to those performed during their first laboratory visit.

To assure compliance (consumption of the supplement/placebo) weekly reminders were made to the cyclists by personal phone calls and by text and e-mail messages. Compliance was 91 %, with 3 dropouts from the E group due to discontinuation of training.

### Supplemented product composition

The probiotic supplement contained about 15 billion colony forming units (CFU) of a probiotic blend consisting of 5 strains: at least (≥) 4.3 × 10^9^ CFU *Lactobacillus helveticus* Lafti L10 (28.6 %), ≥4.3 × 10^9^ CFU *Bifidobacterium animalis* ssp. lactis Lafti B94 (28.6 %), ≥3.9 × 10^9^ CFU *Enterococcus faecium* R0026 (25.7 %), ≥2.1 × 10^9^ CFU *Bifidobacterium longum* R0175 (14.3 %) and ≥0.4 × 10^9^ CFU *Bacillus subtilis* R0179 (2.8 %). Bacteria viability tests were carried out by the manufacturer and the marketing company (Altman Inc. Israel) and clinical documentation from fecal samples presented binding sites were reached [27].The sensorially identical placebo capsules contained the excipients only (potato starch, magnesium stearate, ascorbic acid and white vegetable powder) without the bacteria, which was specially produced for this study by Lallemand Health Solutions Inc. (Montreal, Quebec, Canada) and was identified using random codes for blinding.

### Anthropometric measurements

Body mass (weight), height and body composition were measured before each visit. The cyclists were weighed barefoot, dressed in light underwear using a Shekel model H151-8 scale (Shekel Scales Ltd., Kibbutz Beit Keshet, Israel). Body composition was assessed using Skyndex Electronic Skinfold Caliper (Caldwell, Justiss & Co., Inc., Fayetteville, AR, USA), measuring 4 skinfolds (triceps, biceps, subscapularis, iliac crest) in triplicates and the average of each skin fold was used to calculate body density [28] and percent body fat was calculated using the Siri equation [29]. Body mass index (BMI) was calculated as body mass (kg) / height^2^ (m). All anthropometric measurements were carried out by the same researcher.

### Personal and GI symptoms questionnaire

The online questionnaire was based on Peters et al. [1] questionnaire which was specifically chosen for its unique relevance to endurance athletes. It was administered using “Qualtrics online survey solutions” [30] and consisted of questions referring to socioeconomic status, training, medication, and GI symptoms. GI symptoms prevalence was evaluated during non-exercise periods (e.g., rest), training, competition, and during the first 2 h recovery from training or competition. GI symptoms were classified into UGI tract symptoms (nausea, belching, heartburn, chest pain and vomiting) and the LGI tract (cramps, bloating, diarrhea, flatulence, urge to defecate and defecation). Symptoms incidence was categorized by percentage with a slider questionnaire (0-100 %). Participants indicated the use of liquid or solid food (water, thirst quencher, energy drink, solid food, and/or a homemade product) 2 h before training or competition and during training or competition, categorized as mentioned above. The product names and types of the thirst quenchers, energy drinks and solid foods used had to be indicated, whereas the ingredients of homemade products were also listed. The cyclists were asked about using medication (both for general use, for sports-related symptoms, and for GI symptoms during exercise). The questionnaire was modified and translated from its original English version [1] to the cyclists’ native tongue. Test-retest evaluation of the modified questionnaire was carried out on 12 cyclists who did not participate in the study to assure its consistency with repeated completion after 10 d. Internal reliability values were tested with Pearson correlation and were 0.72, 0.85, 0.72, 0.62 and 0.62 for GI symptoms at rest, at training, at competition, after training and after competition, respectively.

### VO_2_max tests

All exercise tests were performed in an air-conditioned room (24 ± 2 °C, 45 ± 7 % RH) using the same constant-power cycle ergometer (Ergoline Ergometer 100, Bitz, Germany). Saddle height and handlebar reach were measured and documented for each cyclist. Cyclists used their personal pedals and riding shoes and were tested wearing respiratory apparatus and headgear. HR was monitored continuously using a Polar M400 (Polar Electro Finland Oy, Kempele, Finland) telemetry system and was averaged every 5 s during rest and exercise. VO_2_max and other cardio-pulmonary variable (e.g., VTh) were measured using Metalyzer 3B (Cortex Biophysik GmbH, Leipzig, Germany) metabolic cart and were determined following personalized graded exercise protocol. The flowmeter and CO_2_ and O_2_ analyzers were calibrated before each test following the manufacture calibration procedures. After a 10–15 min warmup, the test was conducted beginning at a power output (PO) of ≈ 100 W with personally selected cadence (mean ± SD 90 ± 8 RPM), PO was raised every minute by 25 W until the cyclist reached a volitional exhaustion, his RER (VCO_2_/VO_2_) values were at least 1.15, or his VO_2_ readings did not increase (plateaued) for 3 consecutive 20 s intervals, while PO was raised, or the cyclists asked to stop the test. Typical test duration was 8–13 min. HR was recorded every 20 s and rate of perceived exertion (RPE, scale 6 to 20) was recorded every minute. VTh was determined graphically as the point at which ventilation (V_E_) starts to dramatically increase despite the steady rise in PO and VO_2_, with the ventilatory equivalents method (V_E_/VCO_2_ to V_E_/VO_2_ proportion vs. PO) and was identified at PO in which V_E_/VO_2_ rose while V_E_/VCO_2_ was unchanged or decreased [31, 32].

### Time to fatigue (TTF) test

TTF test was performed 3 h following the conclusion of the VO_2_max test, using the same cycle ergometer with identical individual settings. After 10 min warmup at 50 % of POmax, the cyclists rested for 2 min, thereafter, commenced the TTF test at an intensity of 85 % of POmax. The cyclists were instructed to maintain pedaling cadence at 90–100 RPM, HR was recorded every 15 s and RPE was recorded every minute throughout the test and at the conclusion of the TTF test. TTF was determined when the cyclists’ cadence was lower than 55 RPM. The cyclists neither saw the elapsed time, nor did they receive external encouragement throughout the TTF test.

### Inflammatory markers analysis

Resting venous blood samples were collected into EDTA tubes (BD Vacutainer, Plymouth, UK). Then centrifuged at 1000 g for 15 min at cold (4 °C) centrifuge, the serum was then transferred by Pasteur pipet into 0.5–0.6 ml Eppendorf tubes and was frozen at -40 °C for future analyses. Analyses of C-reactive protein (CRP), IL-6 and TNFα were performed using ELISA kits in accordance with the Quantikine Colorimetric Sandwich ELISA protocol (Minneapolis, MN, USA).

### Statistical analyses

**Fig. 2:**
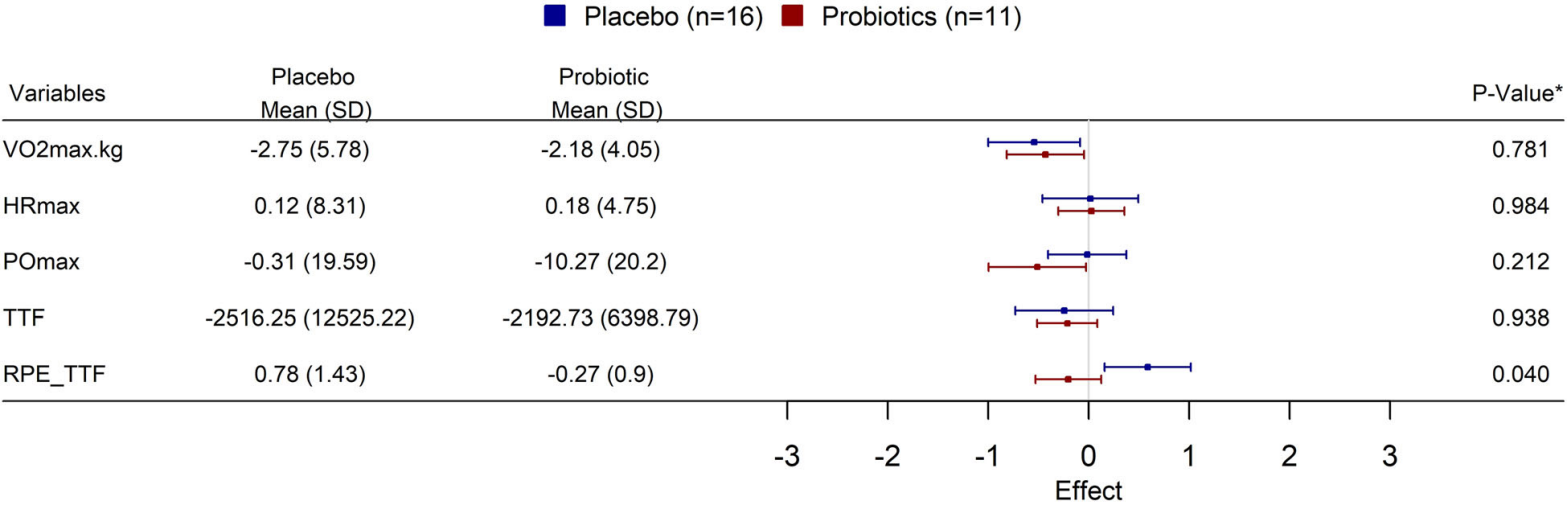
Forest plot describing mean ± SD exercise performance variables’ differences between groups following 3 mo of probiotic supplementation. VO_2_max maximal O_2_ consumption rate (L^.^min^− 1^), HRmax = Heart rate (Beats^.^min^− 1^) at VO_2_max, POmax = maximal power output (W), TTF = Time to fatigue (min:s), RPE_TTF = Rate of perceived exertion at TTF test

**Fig. 3:**
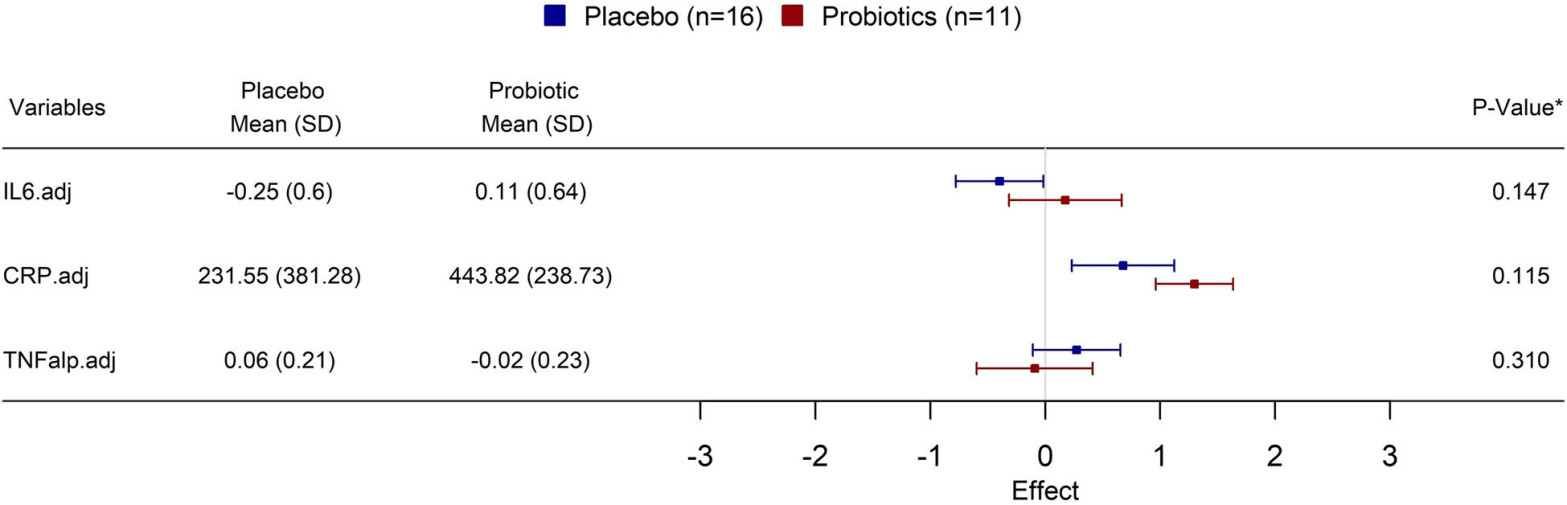
Forest plot describing mean ± SD adjusted (to training duration) inflammation markers’ (cytokines, pg.ml^−^1) differences between groups following 3 mo of probiotic supplementation. IL-6 = interleukin 6, CRP = C reactive protein, TNFalp = Tumor necrosis factor α (TNFα)

**Fig. 4:**
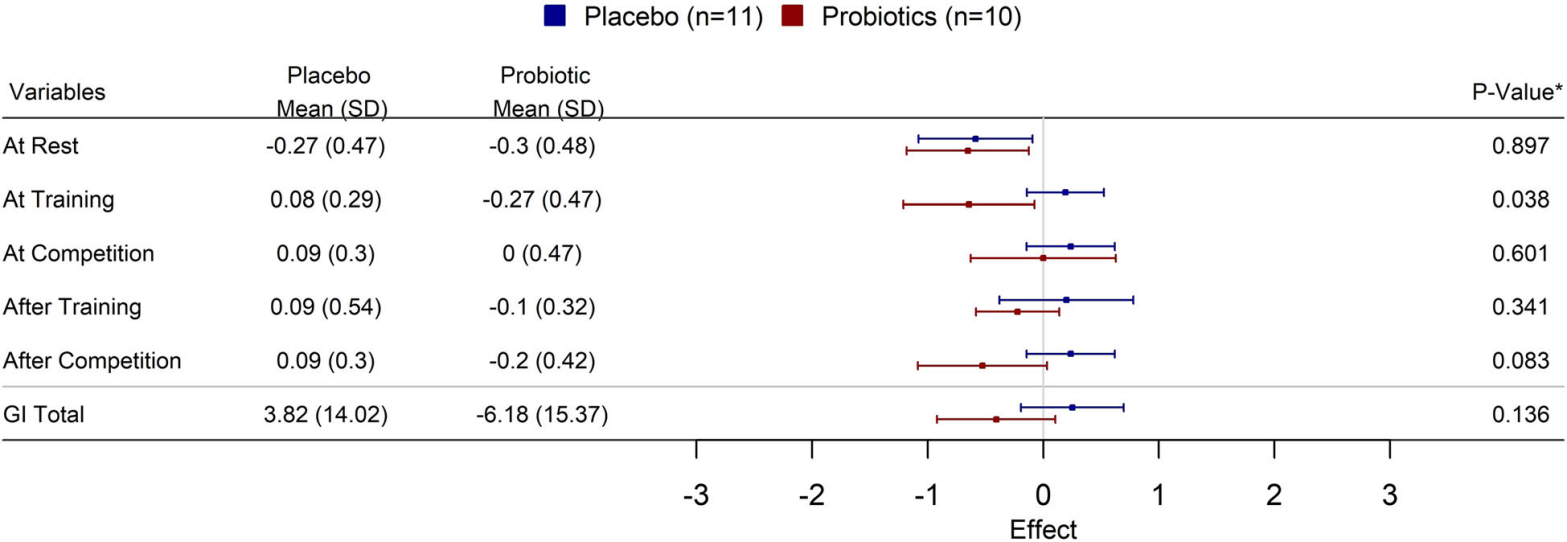
Forest plot describing mean ± SD GI symptoms’ differences between groups following 3 mo of probiotic supplementation

The table-One R package was used to generate results presented as the means ± SD for all variables compared between the intervention groups E vs. C. The data exhibited normal distribution using boxplot analysis and the t-tests were used to compare intervention to placebo. For sensitivity analysis the comparisons were carried-out also with a non-parametric Mann-Whitney test. The non-parametric results were virtually similar to the reported t-test. Effect in each group was calculated as number of SEs below or above the mean group value (Figs. 2, 3 and 4). No corrections for multiple comparisons were done as this was an exploratory pilot study with a small number of participants.

Since the participants’ characteristics analysis revealed difference in training hours during the study period, this was corrected by analysis of covariance (ANCOVA) performed on the main outcomes calculated as delta (Δ) changes from baseline. The adjusted effect size of each dimension (performance, inflammation and GI symptoms) is presented in separate forests-plots to allow graphical evaluation of both general trend of the effects and significant group differences. All analyses were performed using the CRAN R-Project basic, tableOne and Forest plot packages. Significance level was set at *p* < 0.05.

## Results

### Maximal exercise tests

There were no significant differences between E and C groups at baseline in VO_2_max or in any other maximal cardio-pulmonary physiological variables (Table 2) and after 90 d of probiotic supplementation (Fig. 2). Likewise, there were no significant differences between E and C groups in POmax (Fig. 2) or pedaling cadence at VO_2_max or at any other exercise level.

**Table 2:** Fitness characteristics of the participants at the beginning of the study. Data are presented as mean ± SD

	**All Participants (***N*=27**)**	**Experimental** **(***N*=11**)**	**Control** **(***N*=16**)**	*p* value*
**VO**_**2**_**max,** L^.^ min^-1^	4.61±0.45	4.74±0.35	4.51±0.50	0.20
**Specific** **VO**_**2**_**max, **ml^.^min-^1^.kg^-1^	64.7±5.8	66.9±6.4	63.2±5.0	0.10
**HRmax, **min^-1^	183±13	187±15	180±10	0.14

HRmax = maximal heart rate attained at maximal exercise (VO_2_max) test

* *p*<0.05 Experimental vs. Control group (2-tailed independent t test)

### Time to fatigue

No significant treatment effects were found between E and C groups in physiological variables measured during the TTF test (Fig. 2). However, significant changes were found between C and E groups in mean RPE scores following the supplementation period. E group reported a1.3 ± 4.8 % reduction in RPE values while C group reported a4.7 ± 9.7 % increase in RPE (*P* = 0.04, d = 0.91).

### Inflammatory markers

There were no changes in mean adjusted IL-6 (to training duration) (*P* = 0.15, d = 0.57) and CRP (*P* = 0.12, d = 0.73) values and lower mean TNFα values (*P* = 0.31, d = 0.43) in the E group compared with the C group (Fig. 3).

### GI symptoms

Significantly lower incidence of GI symptoms was found during training in the E group compared with the C group (∆GI -27 %±47 % vs.. 8 %±29 %, *P* = 0.04, d = 0.9). Comparison of specific GI symptoms were tested separately, revealing significantly fewer incidences of nausea (∆GI -16 %±43 % vs.. 71 %±119 %, *P* = 0.01, d = 0.9), belching (∆GI -14 %±53 % vs.. 62 %±115 %, *P* = 0.04, d = 1) and vomiting (∆GI -7 %±30 % vs.. 49 %±114 %, *P* = 0.04, d = 0.7) at rest in the E group compared with the C group after 90 d of supplementation. Additionally, there was no between group differences in pooled-overall GI symptoms (*P* = NS, d = 0.6) (Fig. 4).

## Discussion

The main goal of the current research was to study the effect of 90 d multi-strain probiotic supplementation on aerobic fitness characteristics, inflammatory markers values and frequency of GI symptoms among elite road cyclists.

No significant differences were found between the two groups in VO_2_max values following 90 d of probiotics supplementation. These findings are comparable to those reported by Cox et al. [25] who did not find VO_2_max improvement among trained distance runners following 4 wk of supplementation with 1.23 billion CFU of *Lactobacillus fermentum*. It should be noted that although the cyclists in our study were supplemented with probiotics for a longer period and with larger CFU, it did not influence their mean VO_2_max values. There was a high variance (SD > 50 % of the mean value) in some of the exercise performance variables (e.g., HRmax, POmax and TTF).  We speculate that this variability stem from two main sources: the changes between pre and post treatment and the measurement error. Although all participants are professional cyclists, we found substantial variability in their baseline fitness and in their riding routine during the study period.

While the probiotic supplementation was not associated with mean VTh changes of the cyclists, individual changes were noted, and were different between the groups. For example, at least 10 W improvement in PO_VTh_ was observed in 5 cyclists (45 %) of the E group and in 5 cyclists (31 %) of the C group. The lack of significant supplementation effects on performance variables during the TTF test in the current study are in contrast with the results of Shing et al. [6]. This may be due to differences in the environmental conditions (e.g., room vs. hot), the exercise intensity (our cyclists pedaled at 85 % POmax, while in Shing et al.  study participants performed at 80 % of their VTh), and the probiotics dosage (≈15 billion CFU vs. 45 billion CFU).

We observed significant differences in the subjective assessment of the exercise (RPE) before reaching fatigue. The lower RPE values reported by the E group participants during the TTF test may be related to changes in GI symptoms and/or immune system function, which may have influenced their overall subjective feeling during the TTF testing [33, 34]. The TTF durations (< 10 min) in our study were similar to those reported by Cox et al. [25], while Shing et al. [6] measured longer TTF duration (> 30 min) at lower relative PO. Therefore, we speculate that if the TTF test in the current study was performed at a lower intensity, its duration may have been longer with even lower RPE scores reported by the E group. This is supported by the results of Huang et al. [26] study. Although their VO_2_max tests were carried out using a ramp protocol resembling Cox et al. [25] study, their test duration was substantially longer, showing significant results after supplementation in trained triathletes. In a different study, longer TTF values were reported by Huang et al. [24] after probiotics supplementation at similar work intensities used in the current study, however, it is rather difficult to compare their findings to ours, since they performed the TTF test at 85 % of the participants’ VO_2_max values while our cyclists performed the TTF at 85 % of their POmax. Furthermore, Huang et al. [24] participants were not elite athletes and their study investigated the combined effects of exercise training with probiotics supplementation on TTF values.

Mean IL-6, TNFα, and CRP levels were not affected by Probiotic even after adjustment to weekly training durations (h). Our results are inconsistent with those reported by Shing et al. [6] who found no changes in IL-6 in TNFα following 4 weeks of supplementation. These differences may be due to higher workload performed by the cyclists compared to the runners on days preceding testing, although both were instructed to refrain from exercise 24 h prior to testing. Inflammatory marker levels could be affected for several days post exercise, especially after prolonged and multi-day training sessions [35, 36]. For instance, cyclists’ training session duration is 2–4 h compared to shorter training session duration of runners (e.g. 45–90 min). Lamprecht et al. [37] reported a decrease in TNFα following 14 weeks of probiotics supplementation, with no change in IL-6 levels. Their mean baseline TNFα values were clinically higher compared with our values. Furthermore, their athletes were at lower aerobic capacity (mean VO_2_max≈51 ml^.^min^− 1^^.^kg^− 1^) compared with our cyclists (mean VO_2_max≈65 ml^.^min^− 1^^.^kg^− 1^) which may partially explain the disparity in statistical significance between the two studies, as aerobic capacity may affect cytokine regulation. Highly trained athletes are accustomed to greater training loads, thus do not show clinically high TNFα values during periods of intense training, while their IL-6 secretion following exercise might be higher due to longer duration and higher training intensity [16, 36].

Our results on inflammatory markers in cyclists are similar to those reported in previous studies [38]. Fischer [35] found that IL-6 is secreted from exercising muscles (myokine secretion), followed by CRP excretion from the liver. The magnitude of these responses is correlated with the intensity and duration of the exercise. Probiotic supplementation might influence recovery, hence allowing longer and more intense training over time, resulting in elevated IL-6 and CRP levels. To support that, one of the strains used in this study, *L. helveticus Lafti*® L10, showed antioxidant potential, which may affect recovery from exercise [39]. Another explanation may be related to the probiotic effect on gut-associated lymphoid tissue (GALT) resulting in IL-6 production from the gut, as opposed to muscle secretion after exercise [40]. Unlike IL-6, TNFα is only affected by intense and/or prolonged exercise. This might be due to gut endotoxicity caused by LPS entering the blood stream [15, 41]. Future studies should examine the effect of probiotic supplementation on changes in immune markers prior to and following high intensity exercise, to better understand these changes and their link to training load.

The results derived from the GI symptom questionnaire revealed a significant decrease in occurrence of GI symptoms at rest (heartburn, belching and vomiting) and during training (sum of all symptoms) in the E group compared to C group (*P* = 0.04, d = 0.9). These results are in accordance with those published by West et al. [42] who reported a decrease in GI symptom severity after 11 wk of probiotic supplementation. This decrease was more pronounced as exercise intensity increased. This is an interesting finding since these investigators also studied elite cyclists, but without measuring athletic performance [42]. GI symptoms severity after probiotic supplementation was also tested in runners by Shing et al., [6] who found no significant changes in overall GI symptoms severity is similar to our findings. Improvement in GI and upper respiratory symptoms after 28 d of probiotics supplementation was reported in rugby players [43], however, rugby is a high impact sport characterized by short bout efforts, providing different loads on the GI tract and making it an ambiguous comparison to cycling. A recent study reported that 4 wk of probiotic supplementation reduced the incidence and severity of GI symptoms of runners during a marathon race [33]. This finding was correlated with the runners’ ability to maintain pace at the end of the marathon, however there was no effect of probiotic supplementation on their total race time [33]. The results of our study show reduced GI symptom occurrence that might be a result of decreased gut permeability [15, 21]. The effects of probiotic supplementation in our study suggest possible practical application to cyclists and elite endurance athletes. The reduction of GI symptoms incident during training and competition might improve GI function, influencing recovery thus allowing higher training load and volume which may result in improved competitive performance.

## Conclusions

Probiotic supplementation presents an encouraging approach to reduce the incidence and severity of GI symptoms, and RPE of elite endurance athletes (e.g., cyclists) undergoing intense training and competitions. Future research should be carried out using a higher dosage of multi-strain probiotic products. Furthermore, performing similar studies during competitions seasons may lead to tighter control over training phases and may result in meaningful and applicable results. In order to improve our understanding of probiotic effect on gut permeability and endotoxemia, future studies should investigate the effect of intensive exercise on inflammation markers, along with serum LPS levels and other markers of GI permeability. Furthermore, gut microflora and food consumption should be analyzed in order to follow the relationship between changes in gut microflora and athletic performance, thus contribute to better understanding of the effects of probiotic supplementation on symptoms, wellbeing and performance in athletes.

## Study Limitations

Gut flora changes were not analyzed, limiting our understanding of the direct supplementation effects on gut flora. Additionally, FFQ diet questionnaires were not analyzed during the study due to poor participants’ compliance. The cyclists were at various phases of their training/completion season thus, some were at their peak competition level while others were training towards their upcoming competitions season.

## Data Availability

Data may not be shared due to funding company policy which in a process of patent registration of the product.

## Abbreviations

BMI: Body mass index
CFU: Colony forming units
CPET: Cardio-pulmonary exercise test
CRP: C-reactive protein
GI: Gastrointestinal
HR: Heart rate
HRmax: Maximal heart rate attained at VO2 max test
IL-6: Interleukin 6
LGI: Lower gastrointestinal
LPS: Lipopolysaccharide
PO: Power output
POmax: Maximal power outputattained at VO_2_ max test
PO_Vth_: Power outputattained at ventilatory threshold
RER: Respiratory exchange ratio
RPE: Rate of perceived exertion
RPM: Revolutions per minute
TNFα: Tumor necrosis factor alpha
TTF: Time to fatigue
UGI: Upper gastrointestinal
V̇O_2_: Oxygen consumption
V̇O_2_max: Maximal oxygen consumption rate
Vth: Ventilatory Threshold
RH: Relative Humidity

## References

[1] Peters HP, Bos M, Seebregts L, Akkermans LM, van Berge Henegouwen GP, Bol E (1999). Gastrointestinal symptoms in long-distance runners, cyclists, and triathletes: prevalence, medication, and etiology. Am J Gastroenterol.

[2] Lucia A, Hoyos J, Chicharro JL (2001). Physiology of professional road cycling. Sports Med.

[3] Costa RJS, Snipe RMJ, Kitic CM, Gibson PR (2017). Systematic review: exercise-induced gastrointestinal syndrome—implications for health and intestinal disease. Aliment Pharmacol Ther.

[4] De Oliveira EP, Burini RC, Jeukendrup A (2014). Gastrointestinal complaints during exercise: Prevalence, etiology, and nutritional recommendations. Sport Med.

[5] Van Wijck K, Lenaerts K, Grootjans J, Wijnands K, a P, Poeze M, van Loon LJC (2012). Physiology and pathophysiology of splanchnic hypoperfusion and intestinal injury during exercise: strategies for evaluation and prevention. Am J Physiol Gastrointest Liver Physiol.

[6] Shing CM, Peake JM, Lim CL, Briskey D, Walsh NP, Fortes MB (2014). Effects of probiotics supplementation on gastrointestinal permeability, inflammation and exercise performance in the heat. Eur J Appl Physiol.

[7] Miles MP (2020). Probiotics and Gut Health in Athletes. Curr Nutr Rep.

[8] Sánchez B, Delgado S, Blanco-Míguez A, Lourenço A, Gueimonde M, Margolles A (2017). Probiotics, gut microbiota, and their influence on host health and disease. Mol Nutr Food Res.

[9] Calero CDQ, Rincón EO, Marqueta PM (2020). Probiotics, prebiotics and synbiotics: useful for athletes and active individuals? A systematic review. Benef Microbes.

[10] Saunders PU, Pyne DB, Telford RD, Hawley JA (2004). Factors Affecting Running Economy in Trained Distance Runners. Sport Med.

[11] Marttinen M, Ala-Jaakkola R, Laitila A, Lehtinen MJ (2020). Gut microbiota, probiotics and physical performance in athletes and physically active individuals. Nutrients.

[12] Pyne DB, West NP, Cox AJ, Cripps AW (2015). Probiotics supplementation for athletes – Clinical and physiological effects. Eur J Sport Sci.

[13] Hunter CA, Jones SA (2015). IL-6 as a keystone cytokine in health and disease. Nat Immunol.

[14] Ellingsgaard H, Hojman P, Pedersen BK (2019). Exercise and health emerging roles of IL-6. Curr Opin Physiol.

[15] Lamprecht M, Bogner S, Schippinger G, Steinbauer K, Fankhauser F, Hallstroem S (2012). Probiotic supplementation affects markers of intestinal barrier, oxidation, and inflammation in trained men; a randomized, double-blinded, placebo-controlled trial. J Int Soc Sports Nutr.

[16] Pedersen BK (2017). Anti-inflammatory effects of exercise: role in diabetes and cardiovascular disease. Eur J Clin Invest [Internet].

[17] Starkie R, Ostrowski SR, Jauffred S, Febbraio M, Pedersen BK (2003). Exercise and IL-6 infusion inhibit endotoxin-induced TNF-alpha production in humans. FASEB J.

[18] Chelakkot C, Ghim J, Ryu SH (2018). Mechanisms regulating intestinal barrier integrity and its pathological implications. Exp Mol Med.

[19] Jeukendrup AE, Stegen JHJC, Senden J, Saris WHM, Wagenmakers AJM (2000). Relationship between gastro-intestinal complaints and endotoxaemia, cytokine release and the acute-phase reaction during and after a long-distance triathlon in highly trained men. Clin Sci.

[20] Vijayaraghava A, Doreswamy V (2017). Exercise and the cytokines-interleukin-6 (IL-6) and tumor necrosis factor-α (TNF-α): A review. Ann Med Physiol [Internet].

[21] Bron PA, Kleerebezem M, Brummer R-J, Cani PD, Mercenier A, MacDonald TT (2017). Can probiotics modulate human disease by impacting intestinal barrier function?. Br J Nutr.

[22] Colbey C, Cox AJ, Pyne DB, Zhang P, Cripps AW, West NP (2018). Upper Respiratory Symptoms, Gut Health and Mucosal Immunity in Athletes. Sport Med.

[23] Chen Y-M, Wei L, Chiu Y-S, Hsu Y-J, Tsai T-Y, Wang M-F (2016). Lactobacillus plantarum TWK10 Supplementation Improves Exercise Performance and Increases Muscle Mass in Mice. Nutrients.

[24] Huang W-C, Lee M-C, Lee C-C, Ng K-S, Hsu Y-J, Tsai T-Y (2019). Effect of Lactobacillus plantarum TWK10 on Exercise Physiological Adaptation, Performance, and Body Composition in Healthy Humans. Nutrients.

[25] Cox J, Pyne DB, Saunders PU, Fricker PA (2010). Oral administration of the probiotic Lactobacillus fermentum VRI-003 and mucosal immunity in endurance athletes. Br J Sport Med.

[26] Huang W-C, Pan C-H, Wei C-C, Huang H-Y (2020). Lactobacillus plantarum PS128 Improves Physiological Adaptation and Performance in Triathletes through Gut Microbiota Modulation. Nutrients.

[27] Hanifi A, Culpepper T, Mai V, Anand A, Ford AL, Ukhanova M (2015). Evaluation of Bacillus subtilis R0179 on gastrointestinal viability and general wellness: A randomised, double-blind, placebo-controlled trial in healthy adults. Benef Microbes.

[28] Durnin JV, Womersley J (1974). Body fat assessed from total body density and its estimation from skinfold thickness: measurements on 481 men and women aged from 16 to 72 years. Br J Nutr.

[29] Siri WE (1993). Body composition from fluid spaces and density: analysis of methods. 1961. Nutrition.

[30] Qualtrics online survey solutions [Internet]. [cited 2015 Apr 1]. Available from: https://www.qualtrics.com./research-services/.

[31] Caiozzo VJ, Davis JA, Ellis JF, Azus JL, Vandagriff R, Prietto CA (1982). A comparison of gas exchange indices used to detect the anaerobic threshold. J Appl Physiol.

[32] Reinhard U, Müller PH, Schmülling R-M (1979). Determination of Anaerobic Threshold by the Ventilation Equivalent in Normal Individuals. Respiration.

[33] Pugh JN, Sparks AS, Doran DA, Fleming SC, Langan-Evans C, Kirk B (2019). Four weeks of probiotic supplementation reduces GI symptoms during a marathon race. Eur J Appl Physiol.

[34] Halson SL (2014). Monitoring Training Load to Understand Fatigue in Athletes. Sport Med.

[35] Fischer CP (2006). Interleukin-6 in acute exercise and training: What is the biological relevance?. Exerc Immunol Rev.

[36] Peake JM, Della Gatta P, Suzuki K, Nieman DC (2015). Cytokine expression and secretion by skeletal muscle cells: regulatory mechanisms and exercise effects. Exerc Immunol Rev.

[37] Lamprecht M, Bogner S, Schippinger G, Steinbauer K, Fankhauser F, Hallstroem S (2012). Probiotic supplementation affects markers of intestinal barrier, oxidation, and inflammation in trained men; a randomized, double-blinded, placebo-controlled trial. J Int Soc Sports Nutr.

[38] Kasapis C, Thompson PD (2005). The Effects of Physical Activity on Serum C-Reactive Protein and Inflammatory Markers A Systematic Review. J Am Coll Cardiol.

[39] Michalickova D, Kotur-Stevuljevic J, Miljkovic M, Dikic N, Kostic-Vucicevic M, Andjelkovic M (2018). Effects of Probiotic Supplementation on Selected Parameters of Blood Prooxidant-Antioxidant Balance in Elite Athletes: A Double‐Blind Randomized Placebo‐Controlled Study. J Hum Kinet.

[40] West NP, Pyne DB, Peake JM, Cripps AW (2009). Probiotics, immunity and exercise: A review. Exerc Immunol Rev.

[41] Al-Sadi R, Guo S, Ye D, Rawat M, Ma TY (2016). TNF-α Modulation of Intestinal Tight Junction Permeability Is Mediated by NIK/IKK-α Axis Activation of the Canonical NF-κB Pathway. Am J Pathol.

[42] West NP, Pyne DB, Cripps AW, Hopkins WG, Eskesen DC, Jairath A (2011). Lactobacillus fermentum (PCC®) supplementation and gastrointestinal and respiratory-tract illness symptoms: a randomised control trial in athletes. Nutr J.

[43] Haywood BA, Black KE, Baker D, McGarvey J, Healey P, Brown RC (2017). Probiotic supplementation reduces the duration and incidence of infections but not severity in elite rugby union players. J Sci Med Sport.

